# Supplementary material for: Sequesterpene Lactones Isolated from a Brazilian Cerrado Plant (Eremanthus spp.) as Anti-Proliferative Compounds, Characterized by Functional and Proteomic Analysis, Are Candidates for New Therapeutics in Glioblastoma
Source: Int J Mol Sci. 2020 Jul 1;21(13):4713. doi: 10.3390/ijms21134713 (PMC7369765; doi:10.3390/ijms21134713)
Supplement: Supplementary file 1 [file ijms-21-04713-s001.zip › Supp Fig 1.docx]

Evaluation of viability of MDCK cells treated with sesquiterpene lactones AM04 (goyazensolide) and AM05 (lychnofolide), and temozolomide (TMZ).


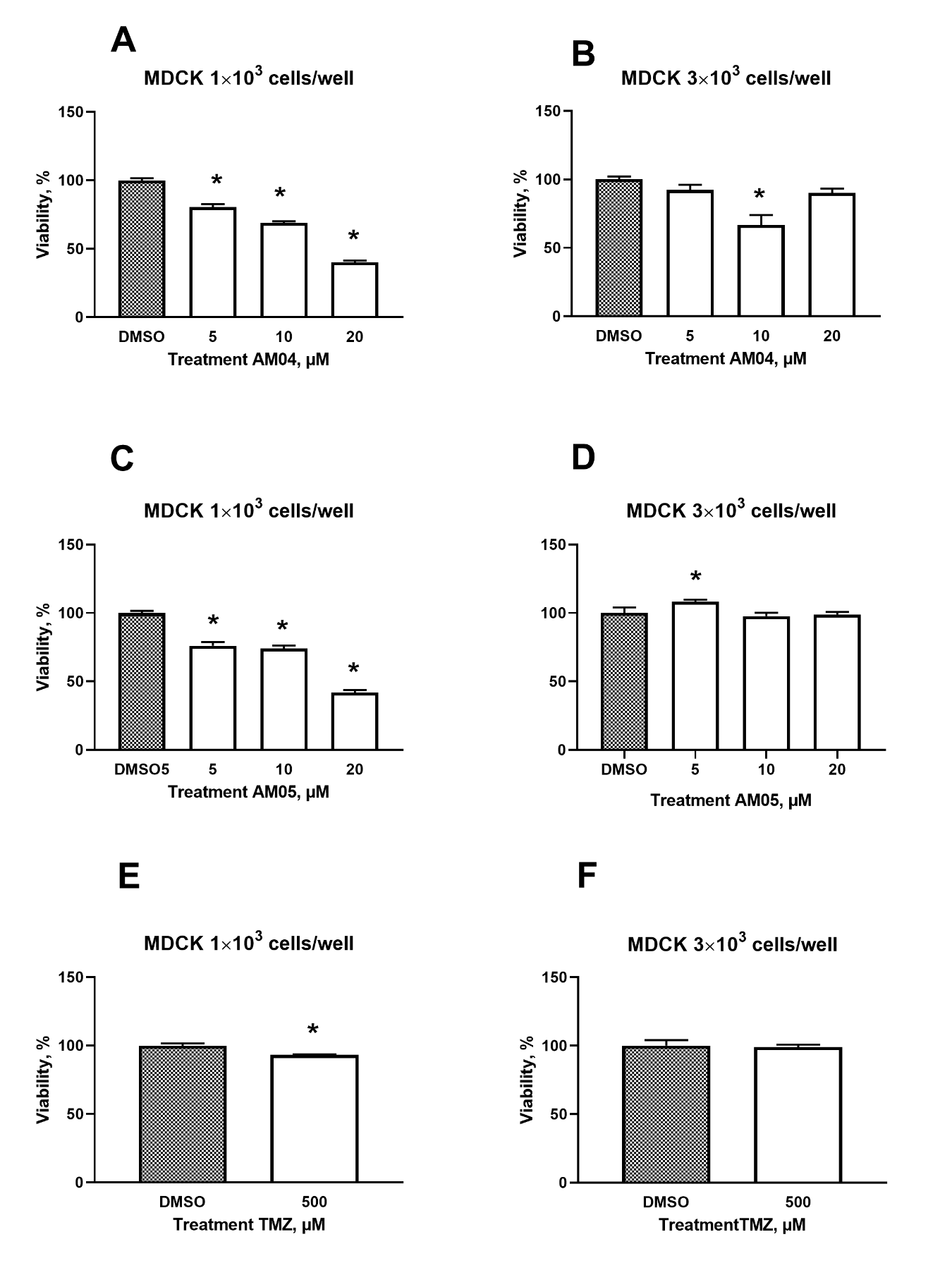


Figure S1 – Evaluation of viability of MDCK cells treated with sesquiterpene lactones AM04 (goyazensolide) and AM05 (lychnofolide), and temozolomide (TMZ). MDCK cells were seeded in 96 well plates in concentration of 1×10^3^ cells/well (Fig 1SA, 1SC, and 1SE) and 3×10^3^ cells/well (Fig S1B, S1D and S1F). After overnight period for adherence to the bottom of wells, the cells were treated with the different drugs for additional 24h (AM04 and AM05 at 5.0, 10.0, and 20.0 µM, and TMZ at 500 µM). The viability was determined by XTT reaction. Results were expressed as Mean±SEM, n=4. For comparative analysis of AM04 and AM05 groups of data, one-way ANOVA was used, followed by Dunnett’s multiple comparisons test (*p<0.0001, compared to DMSO, for Fig 1SA, 1SC; p=0.0006, compared to DMSO for Fig 1SB, and p=0.0026 for Fig 1SD). The TMZ treatments were analyzed by unpaired t-test (*, p<0.05 for Fig 1SE).

Figure S1 demonstrates that at lower quantity of cells, 1×10^3^ cells/well, both AM04 (Fig S1A) and AM05 (Fig SC), induced a significantly reduction on cell viability of MDCK cells in all concentrations used. The same effect was observed for treatment with 500 µM TMZ (Fig S1E) At 3×10^3^ cells/well, AM04 reduced slightly the cell viability of MDCK cells at 10 µM (p=0.0006) but not affected the cell viability at 20 µM; however, AM05 and TMZ did not affected the MDCK cell viability at this quantity of cells. Thus, these results allowed the study of membrane permeability using MDCK cells and these drugs.
