# Supplementary figures and images for: Sequesterpene Lactones Isolated from a Brazilian Cerrado Plant (Eremanthus spp.) as Anti-Proliferative Compounds, Characterized by Functional and Proteomic Analysis, Are Candidates for New Therapeutics in Glioblastoma
Source: Int J Mol Sci. 2020 Jul 1;21(13):4713. doi: 10.3390/ijms21134713 (PMC7369765; doi:10.3390/ijms21134713)

## Slide 1
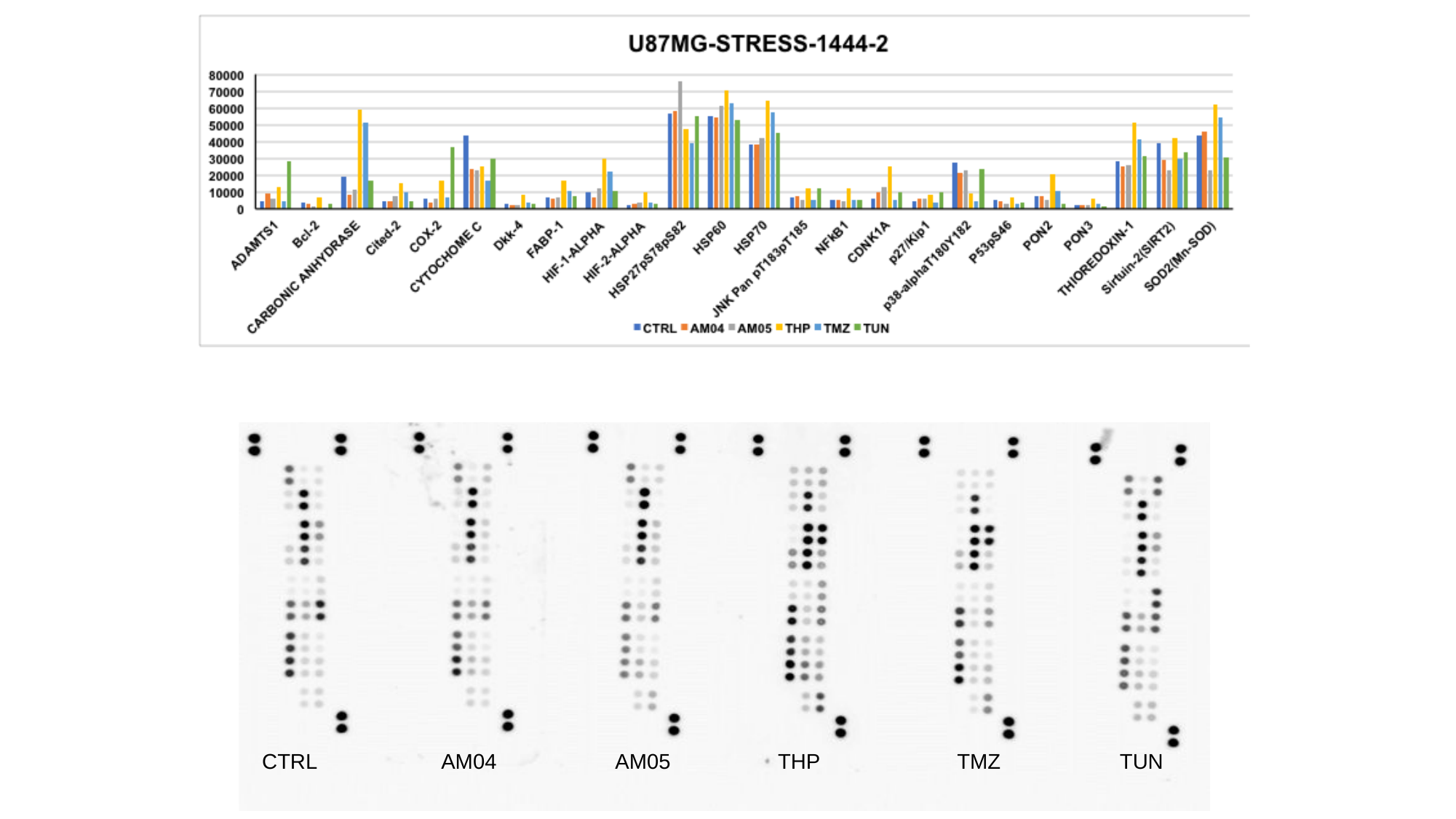

CTRL AM04 AM05 THP TMZ TUN

Supplement: Supplementary file 1 [file ijms-21-04713-s001.zip › Supp Fig 5 U87MG stress arrray.pptx]

## Slide 1
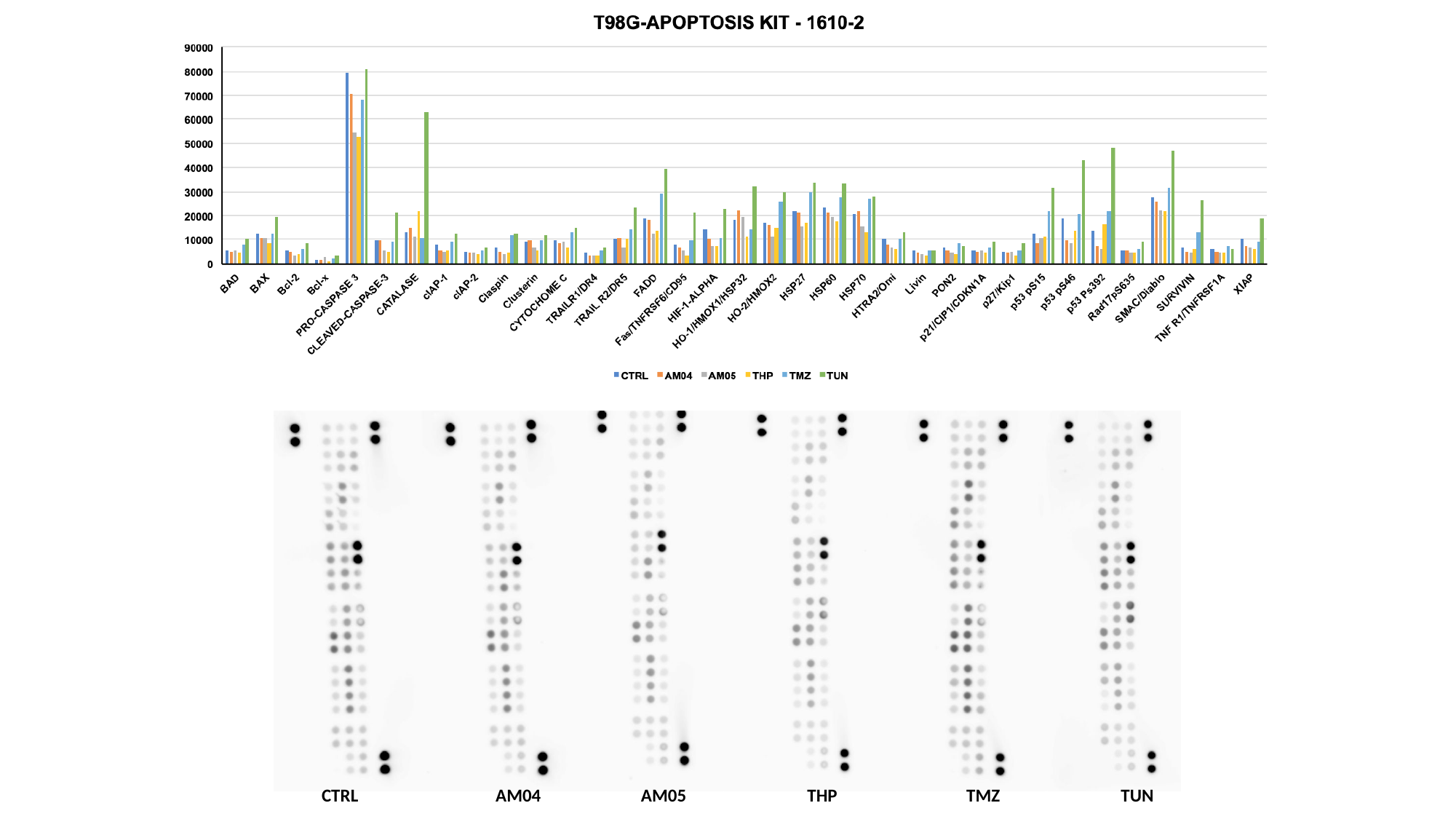

CTRL AM04 AM05 THP TMZ TUN

Supplement: Supplementary file 1 [file ijms-21-04713-s001.zip › Supp Fig 2 T98G apoptosis arrray.pptx]

## Slide 1
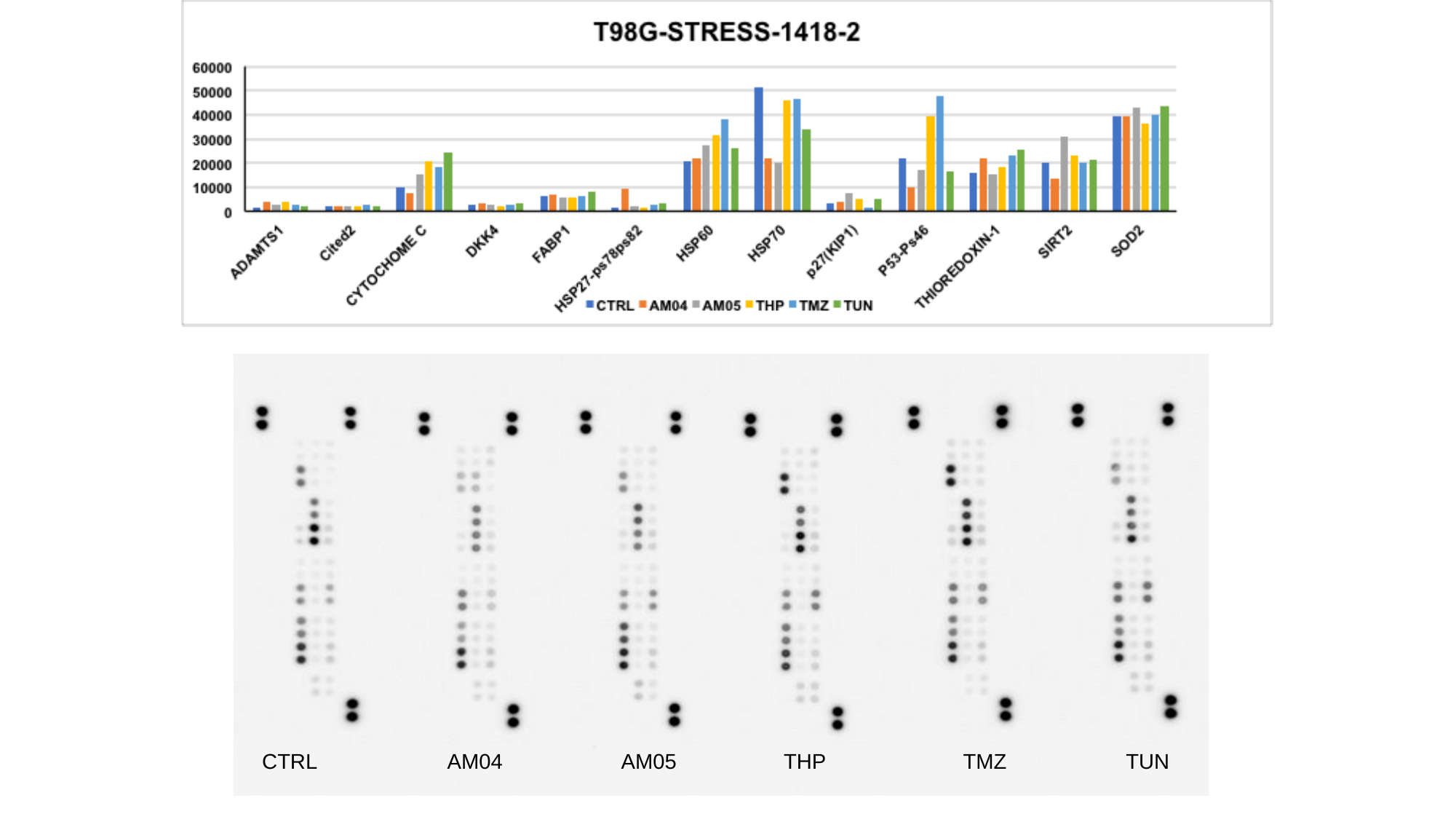

CTRL AM04 AM05 THP TMZ TUN

Supplement: Supplementary file 1 [file ijms-21-04713-s001.zip › Supp Fig 3 T98G stress arrray.pptx]

## Slide 1
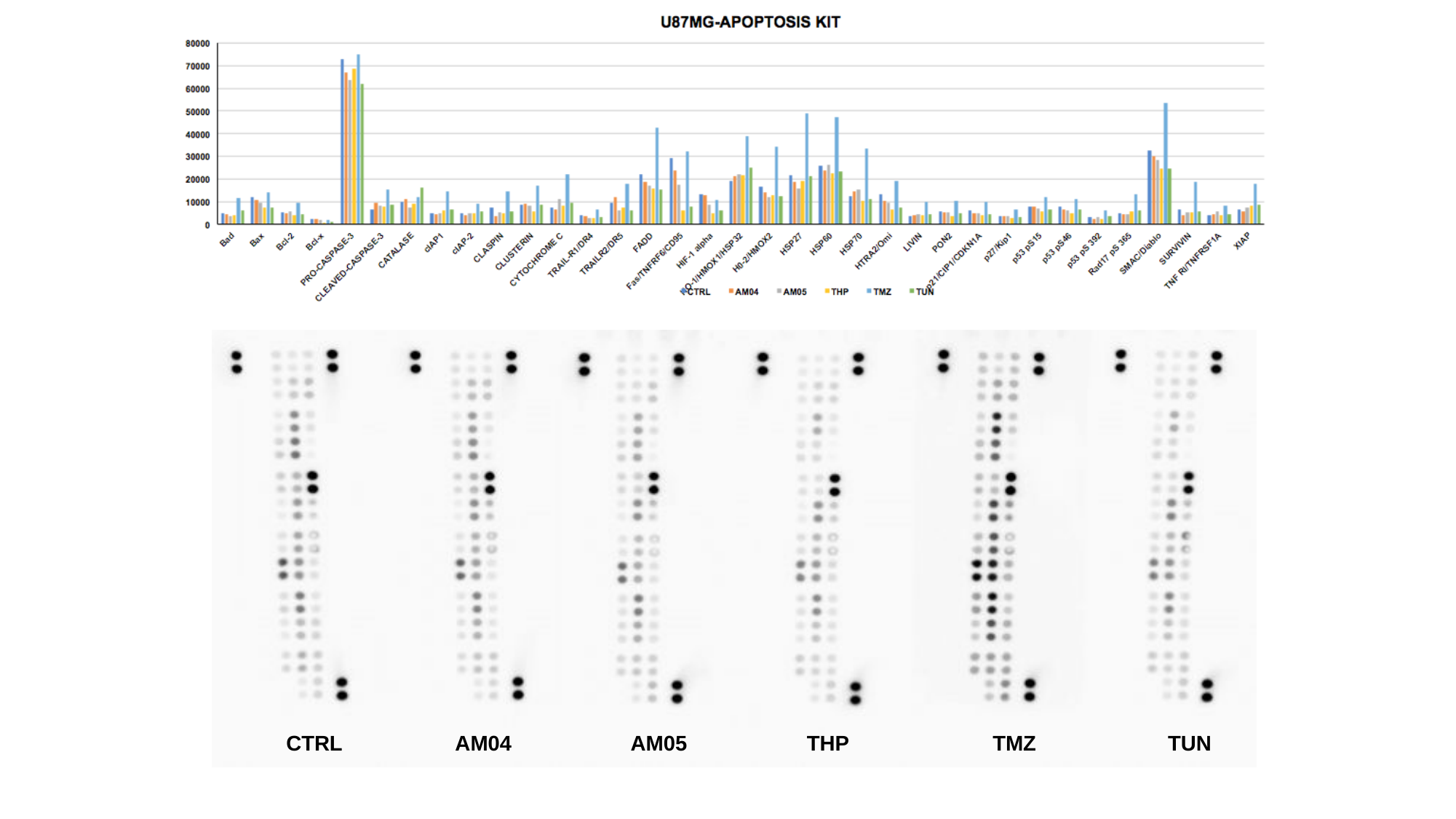

CTRL AM04 AM05 THP TMZ TUN

Supplement: Supplementary file 1 [file ijms-21-04713-s001.zip › Supp Fig 4 U87MG apoptosis arrray.pptx]
